# Effect of Nanotube Film Thickness on the Performance of Nanotube-Silicon Hybrid Solar Cells

**DOI:** 10.3390/nano3040655

**Published:** 2013-12-17

**Authors:** Daniel D. Tune, Joseph G. Shapter

**Affiliations:** School of Chemical and Physical Sciences and Flinders Centre for Nanoscale Science and Technology, Flinders University, GPO Box 2100, Adelaide 5001, Australia; E-Mail: daniel.tune@flinders.edu.au

**Keywords:** carbon nanotubes, hybrid solar cells, layer thickness, efficiency

## Abstract

The results of measurements on solar cells made from randomly aligned thin films of single walled carbon nanotubes (SWCNTs) on *n*-type monocrystalline silicon are presented. The films are made by vacuum filtration from aqueous TritonX-100 suspensions of large diameter arc-discharge SWCNTs. The dependence of the solar cell performance on the thickness of the SWCNT film is shown in detail, as is the variation in performance due to doping of the SWCNT film with SOCl_2_.

## 1. Introduction

There is a clear need to make energy cheap, readily accessible and green, while ensuring its production does not contribute to further climate change. Of all the options available, photovoltaics offer the highest probability of delivering a meaningful and sustainable change in the way society produces its energy. Two key factors in the ongoing technological advancement of photovoltaics are that the average daily global solar irradiance is more than enough to satisfy current and future energy needs and that a move away from the current grid distribution system will see an immediate improvement in energy use efficiency on the order of 25% [1]. This is because, in the current system, about a quarter of all electricity produced is lost in distribution. A system where electricity is created where needed would be far better and photovoltaics offer this opportunity. Other solar solutions such as solar-thermal can be significantly more expensive to implement, both upfront and in the long term, and are subject to the same transmission problems [1]. Meanwhile, many photovoltaic systems suffer critical drawbacks such as the toxicity or scarcity of input materials including Cd, In, Ga and Ru, or by short product lifetimes.

Thus, there is currently great interest in finding alternative materials and/or alternative ways of fabricating the current generation of solar cells. One material with great potential in these areas is carbon, in the form of carbon nanotubes. Carbon is certainly not in limited supply and this material could be made very, very cheap with economies of scale in production. Carbon nanotube-silicon solar cells are a recent photovoltaic system [2,3,4]. A typical device has an architecture similar to that of a single junction monocrystalline *n*-type silicon solar cell with the exception that the *p*-type emitter is replaced by a thin film of carbon nanotubes. The exact mechanism of operation of such cells is still under debate, with some reports suggesting a *p*-*n* junction-like mechanism and others supporting a Schottky junction mechanism. Confusion may be arising due the diversity and complexity of nanotube films used, as well as the preparation methods and any treatments applied during fabrication. Additionally, single walled carbon nanotubes (SWCNTs) can be either metallic or semiconducting and most reports have used unsorted mixtures. However, huge differences are observed between devices fabricated with nanotubes sorted into highly pure metallic or semiconducting fractions, with or without subsequent chemical doping of the nanotubes and it may be that different mechanisms apply depending on the particular kind of device in question [5].

There has been considerable work already in the field of carbon nanotube-silicon solar cells. Several reports deal in part with the electrical and optical characteristics of the nanotube film [6,7,8,9,10,11,12,13]. The first report of carbon nanotube-silicon solar cells appeared in 2007 with Wei *et al.* [6] who demonstrated a 1.3% efficient device. In that double walled carbon nanotubes (DWCNTs) were deposited on *n*-type silicon substrates via H_2_O expansion and subsequent aqueous film transfer of an as-grown chemical vapor deposition (CVD) film. Jia *et al.* [14] reported a significant step forward from the 2007 work upon demonstrating a 7.4% efficient DWCNT-silicon device. Subsequently, other groups have used alternative methods to deposit thin films of SWCNTs onto *n*-type silicon. Li *et al.* [7,8,9] reported a SWCNT-silicon solar cell employing a SWCNT film spray coated using an airbrushing technique from a dimethylformamide (DMF) suspension. Final devices were found to afford an efficiency of 1.3%. Li *et al.* [7] also investigated SWCNT film post-treatment methods in order to increase device efficiency. Hall Effect measurements showed that post-treatment of the SWCNT film with thionyl chloride (SOCl_2_) leads to increases in carrier density and effective mobility.

Jia *et al.* [9] used the SWCNT deposition method of Wu *et al.* [15], which employs vacuum filtration onto mixed cellulose ester (MCE) films with subsequent removal of the MCE by dissolving in acetone. The MCE deposition method was used for SWCNTs and multiwalled carbon nanotubes MWCNTs but aqueous film transfer of a self-assembled film (as per their prior work) [6,14] was used for DWCNTs. It was found that SWCNT films outperform MWCNTs only when the density is low, which is interpreted as indicating that optical transmittance is the most important variable in comparing SWCNTs and MWCNTs. The authors also correlate a figure of merit (*FM*) for transparent, conductive films with the power conversion efficiency (PCE) of carbon nanotube-silicon solar cells such that *FM* = *T*_550_ (%)/*R*_sheet_ (Ω·sq^−1^) where *T*_550_ is the optical transmittance for λ = 550 nm and *R*_sheet_ is the sheet resistance. It was found that the *FM* is proportional to PCE such that increasing film transparency or decreasing sheet resistance yields higher PCE. Optical transparency is increased for thinner films whereas the sheet resistance is decreased for thicker films. Thus at some point there must be a trade-off between these two variables with the optimal thickness likely constrained by the optimization of other cell parameters. The effect of nanotube film thickness has also been investigated by Castrucci *et al.* [16] who similarly found that the density of the nanotube film (number of nanotube-silicon junctions) is a vital parameter in optimizing performance.

Wadhwa *et al.* [17] report a novel method of improving SWCNT-silicon solar cells through electronic junction control of a SWCNT-silicon device by the use of a gate potential applied to the junction via the ionic liquid electrolyte 1-ethyl-3-methylimidazolium bis(trifluoromethylsulfonyl)imide (EMI-BTI). The device exhibited a PCE of 8.5%, which was dynamically and reversibly adjusted to between 4% and 11% by electronic gating. The mechanism of action of the electronic junction control is explained by considering the gate-induced modulation of the SWCNT Fermi level and the gate-modulated enhancement or suppression of the interface dipole at the junction. Wadhwa *et al.* [18] extend previously reported work by engineering a grid pattern in the SWCNT layer through oxygen plasma etching. A modest improvement in PCE from ~11% to ~12% is observed via this strategy.

Jia *et al.* [19] achieved a much higher PCE of 13.8% by *in situ* doping of the SWCNT film with 0.5 M HNO_3_ although, as shown in the later work of Jung [20], this may have been partly due to lensing by the acid droplet. The untreated device exhibited a PCE of 6.2% and the improvement was due to an increase in short circuit current density (*J*_sc_) from 27 mA·cm^−2^ to 36 mA·cm^−2^ coupled with an increase in the fill factor (*FF*) from 0.47 to 0.72. Even after drying of the acid solution, the cell maintained a higher PCE than the original. In another work by Jia *et al.* [21], encapsulation of the active area by the insulating polymer polydimethylsiloxane (PDMS) was shown to increase PCE whilst providing greatly improved device stability. Additionally, it was shown that the formation of a thin SiO*_x_* layer in between the SWCNTs and the underlying silicon leads to gains in open circuit voltage (*V*_oc_).

It is clear that carbon nanotube films function, at least, as transparent conducting front electrodes. Carbon nanotube films have been used similarly in amorphous silicon solar cells [11,22], heterojunction solar cells of quantum dots and silicon [23,24], on silicon nanowires with [25] and without [26,27] photoactive polymers, as well as enhancing the performance of “standard” *p*-*n* junction silicon solar cells [28]. Photovoltaic output from SWCNT-silicon cells can be improved by the addition of graphene “patches” to the nanotube film [29] and by flowing gasses over the surface of the nanotube film, an effect that has been used to fabricate gas sensors [30]. A recent development is the use of highly aligned SWCNT films [20,31]. Devices made with such films show good performance, likely due to the greatly improved electrical characteristics of aligned SWCNT films. In the work by Shi *et al.* [32], the use of a TiO_2_ antireflection layer has been reported to yield SWCNT-silicon solar cells with a PCE of 15% which puts them into the same region of PCE as many commercial solar panels.

Because of the large variety of SWCNT films reported in the literature, the methods of their preparation, deposition and treatment, as well as physical, electrical and optical characteristics, it can sometimes be difficult to ensure the validity of comparisons between different devices. The work in this paper uses a vacuum filtration method throughout to maintain test consistency and allow less ambiguous comparison between results. As in other reports, the performance of SWCNT-silicon solar cells fabricated with such films will be shown to depend very strongly on the optical density and conductivity of the film. In the simplest sense, the best films are those with both the highest transmittance and lowest resistance however there is often a trade-off needed in the assessment of film quality.

## 2. Results and Discussion

Vacuum filtration films of SWCNTs were used to fabricate solar cells and also deposited on glass to allow optical characterization and measurement of the sheet resistance. Additionally, nine different optical densities of film were made by using varying volumes of SWCNT suspension. Each complete solar cell was made in duplicate and solar cell parameters are averaged from both devices with the standard deviation displayed by error bars in the plotted data.

### 2.1. SWCNT Film Thickness

**Figure 1:**
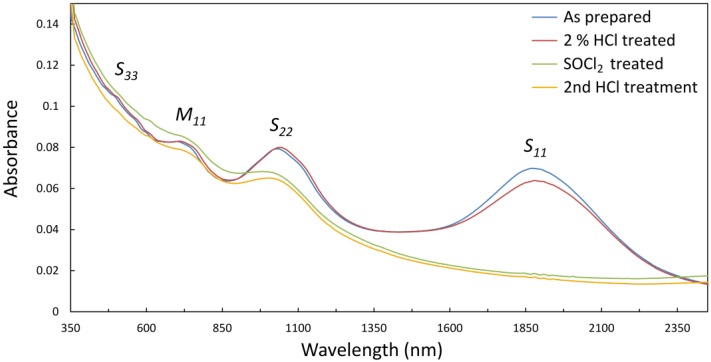
UV-vis-NIR spectra of thin vacuum filtration films of single walled carbon nanotubes (SWCNTs) on glass, made from aqueous TritonX-100 suspensions of large diameter arc-discharge material.

The ultraviolet-visible-near-infrared (UV-vis-NIR) optical absorption spectrum of an as prepared film of arc-discharge SWCNTs is shown in Figure 1. The low energy of the S_11_ transition (~0.7 eV) is consistent with large diameter SWCNTs and the broadness of the absorption features is indicative of both the polychirality of the material and the bundled state of the dispersion [33]. Following treatment with 2% HCl (used *in lieu* of hydrofluoric acid (HF) for the optical measurement of films on glass—see experimental method) the S_11_ absorption peak is slightly suppressed. This is expected due to mild *p*-type doping of the SWCNTs by transfer of charge during protonation by the acid. Following treatment with SOCl_2_ there is complete bleaching of the S_11_ absorption and a substantial decrease in the intensity of S_22_, but an overall absorption increase for λ < 1100 nm. Bleaching of S_11_ is consistent with electron transfer from the top of the SWCNT valance band to the organic oxidizer as previously observed [34,35]. The origin of the increase in the lower λ region is unknown and it was not always observed, but was always removed by the second acid treatment and may have been due to incomplete removal of SOCl_2_ decomposition products. Importantly, there was no return of S_11_ after the second acid treatment.

**Figure 2:**
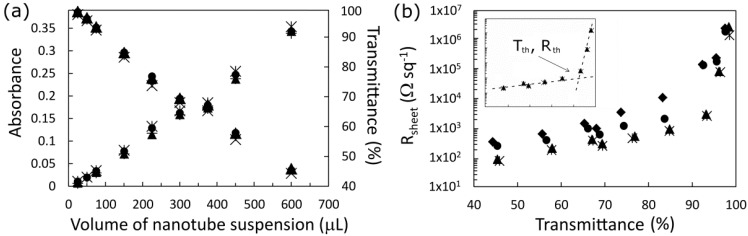
(**a**) Dependence of the SWCNT films’ average optical depth on the volume of SWCNT suspension used per square centimeter of filtration membrane. The different symbols represent different treatments and show very that there is very little effect on the baseline visible absorption; (**b**) Variation of the sheet resistance with film transmission for as prepared (♦), HCl treated (●), SOCl_2_ treated (■) and HCl retreated (▲) devices. Inset shows the two regions of differing resistance for the doped films and the threshold transmittance (*T*_th_) and corresponding sheet resistance (*R*_th_).

The linear relationship between absorbance and volume of SWCNT suspension shown in Figure 2a is expected from Beer’s law where the absorbance *A* is proportional to *N*, the number of absorbing units [36]. Although there is a logarithmic relationship between absorbance and transmittance, *T*_av_ is in the range of relative linearity (99% > *T* > 40%) with *N*. The very small difference between average absorbance before and after the various treatments is an indicator of the efficacy of the averaging process in removing the effect of the absorption peaks.

In Figure 2b, the plot of sheet resistance, *R*_sheet_, *versus* film thickness (expressed here as *T*_av_) shows the same apparent exponential increase seen as in prior reports [8,37]. *R*_sheet_ is composed of two regions of resistance: one (*R*_junction_) where conductance is limited by the number of tube-tube junctions and their contact resistance, and the other (*R*_tube_) limited by the concentration of carriers and their mobility, such that; *R*_sheet_ = *R*_junction_ + *R*_tube_. The *R*_tube_ term dominates for the thicker films, which are more metal-like, whilst *R*_junction_ dominates for the sparsest films in which transport is governed by percolation theory [37,38,39,40]. The point *T*_th_ is the threshold of transmittance/thickness/density at which the conductance of the film switches from being dominated by percolation to a more bulk metallic behavior. *T*_th_ is a figure of merit for these films, with higher values indicating a better electrode film, and its effect will be clearly seen in the solar cell performance measurements that follow. *R*_th_ is *R*_sheet_ at *T*_th_ and clearly, the lower *R*_th_ is the better. Even though the acid treatment had little effect on the optical absorbance, *T*_th_ increased markedly from ~65% to ~85% when the SWCNT films on glass were exposed briefly to 2% HCl, and this was coupled with an order of magnitude decrease in *R*_th_. This initial improvement is likely due to removal of residual surfactant molecules by the acid. Another big increase in *T*_th_ up to 90%–95% is observed following the SOCl_2_ treatment, as well as large reductions in *R*_sheet_ in the range 45%–85%. However no further improvements are seen following a second 2% HCl treatment, indicating that any improvements observed in solar cell devices from the second HF treatment are due to the effect it has on the silicon.

### 2.2. As-Prepared Solar Cells

**Figure 3:**
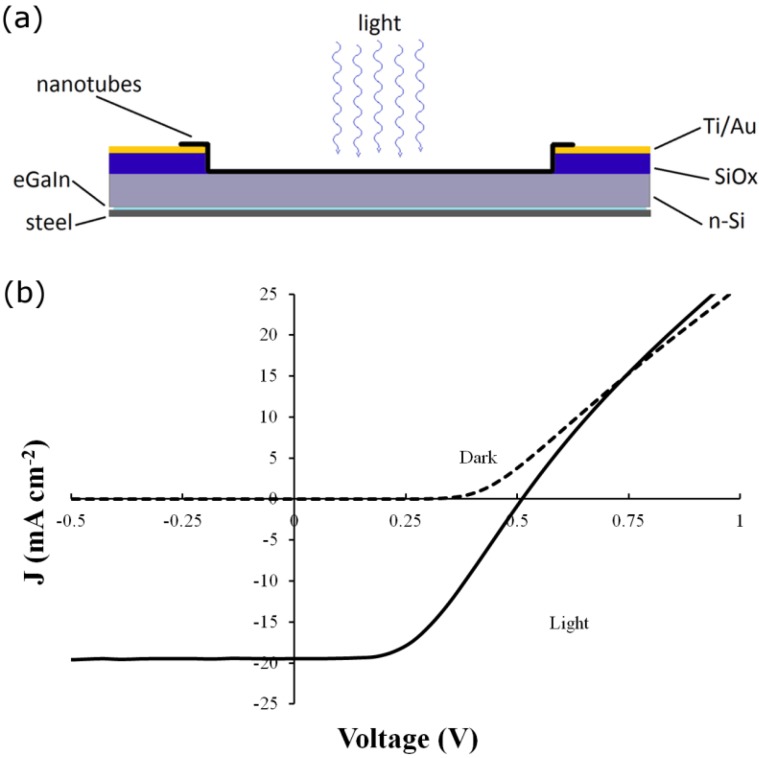
(**a**) Device schematic; (**b**) JV curve of a typical SWCNT-Si solar cell.

A typical JV curve for a single-walled carbon nanotube-silicon hybrid solar cell is shown in Figure 3.

**Figure 4:**
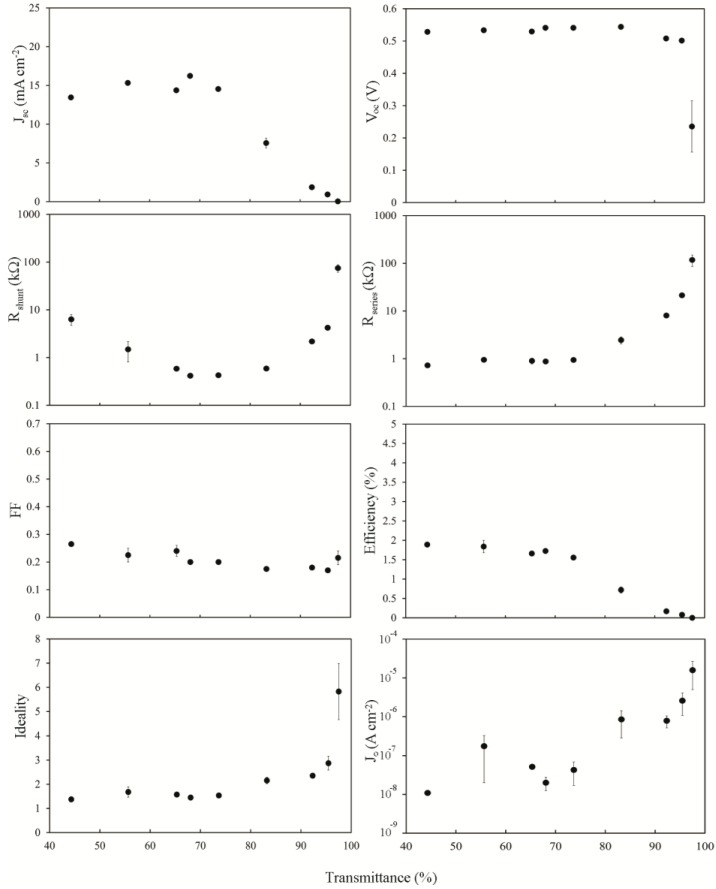
Solar cell parameters extracted from as-prepared SWCNT-Si devices with varying SWCNT film thickness.

The light and dark current-voltage characteristics of the as-prepared devices were measured as a function of the optical density, or thickness, of the SWCNT film and are summarized in Figure 4. The peak in short circuit current density, *J*_sc_, around *T*_av_~60%–70%, occurs due to a balancing of the decrease in the photon flux, *J*_ph_, penetrating through to the silicon for the thicker films with *T*_av_ < 65%, and the steep exponential rise in *R*_sheet_ above *T*_th_. In fact, the same pattern of variation seen in *R*_sheet_ is exactly replicated here in the plot of series resistance, *R*_s_, for films with *T*_av_ > 75%. The open circuit voltage, *V*_oc_, is remarkably consistent across the thickness range until the thinnest film with *T*_av_ = 97%. There is a clear but modest decrease in *FFs* towards thinner films. There is also a noticeable U-shape observed in the plot of the shunt resistance, *R*_shunt_. This can be easily explained for *T*_av_ > 75% as it exactly mirrors the pattern of variation evident in *R*_sheet_, *R*_s_ and PCE, and thus may be similarly due to increasingly limited percolation pathways. For *T*_av_ < 75% the cause of the increase in *R*_shunt_ is less obvious, but could be due to a better depletion region set up in the silicon as a result of the higher total carrier concentration in the thicker films. Li [8] and Kozawa [12] observed a peak in the performance of their SWCNT-silicon solar cells at ~60% film transmittance, likely related to *T*_th_. However, in other work performance kept increasing for increased transmittance up to the maximum used in that experiment (90.4%) [10] likely indicating that *T*_th_ > 90.5% for those films. The reverse saturation current density, *J*_0_, is good (low) for the thickest films at ~10^−8^ A·cm^−2^ but is also clearly dependent on the SWCNT film thickness.

Overall efficiency shows the same two distinct regions as seen in *R*_sheet_, *J*_sc_, *R*_s_ and part of *R*_shunt_, and strongly implicates the poor sheet resistance of the SWCNT films as the major limiting factor underlying the poor performance of all the devices measured (<2%). When considering that *R*_s_ of these devices was, at best, 1 kΩ, and that the typical area normalized series resistance of commercial cells can range from 0.5 to 2 Ω·cm^2^, that is not really surprising.

### 2.3. The Effect of HF Treatment

The purpose of the HF treatment is to remove the relatively thick oxide present on the silicon surface between the silicon and the SWCNTs. This oxide would have likely been thickened somewhat during the wet and hot film deposition process. Measurements were not taken immediately following the HF treatment due to the observation of unpredictable and random results with large variations in output, sometimes even between consecutive measurements of the same device. Unlike the perfect silicon hydride that can be formed on an uncovered surface, the presence of the SWCNTs on these surfaces likely contributes to the formation of dangling bonds and trap states during the HF treatment. Measurements were observed to stabilize after an hour in air at room temperature (not long enough for the formation of an intermediate oxide >1 nm).

**Figure 5:**
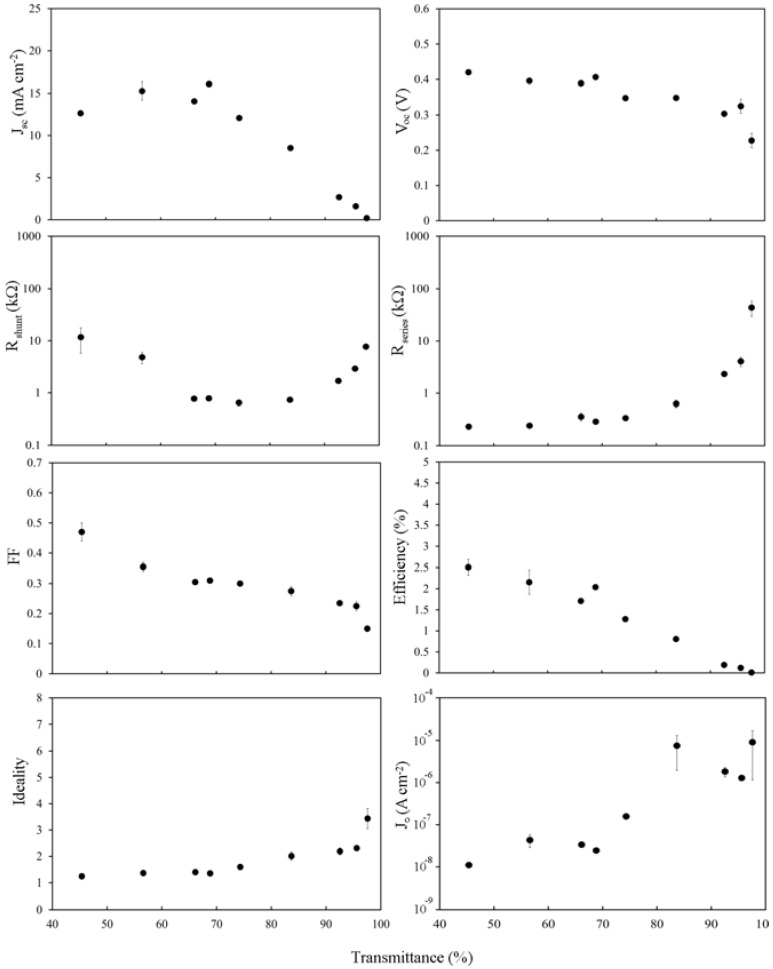
Solar cell parameters extracted from HF treated SWCNT-Si devices with varying SWCNT film thickness, 1 h after treatment.

Immediately apparent when comparing Figure 4 and Figure 5 is that *J*_sc_ is almost entirely unchanged, consistent with *J*_sc_ being dependent primarily on *J*_ph_, which is not changed significantly by brief exposure to mild acid, and on the shape of *R*_s_. Importantly, *V*_oc_ now shows a definite dependence on the SWCNT film thickness and has been reduced by >10% in all cases. The fill factor has been improved; doubling for thicker films, whilst also now showing a stronger dependence on the film thickness than before. Improvements in *FFs* give an increase in the highest PCE from 1.8% to 2.5%, although other factors combine to decrease PCE much faster with reducing film thickness. The effect of lower *R*_sheet_, particularly in the range of 60% < *T*_av_ < 90%, is seen in the plot of *R*_s_ however there is little change to *R*_shunt_ or *J*_0_.

Overall, it appears that the rapid increase in *R*_sheet_ above *T*_th_ dominates all measures of the device IV characteristics for *T*_av_ > 70%, and this occurs via its effect on *R*_s_. Such strong dependence supports the hypothesis that, in these devices, the role of the SWCNT film is as a transparent, conducting front electrode that must also (clearly) establish an inversion layer/depletion region in the underlying silicon.

### 2.4. The Effect of SOCl_2_ Treatment

A common method to reversibly improve the electrical properties of SWCNT films is to expose them to the powerful organic oxidizer, SOCl_2_ [35,41,42,43,44,45,46]. This has the effect of hole doping the SWCNTs via the electron withdrawing nature of the decomposition reaction:

2SOCl_2_ + 4e^-^ → S + SO_2_ + 4Cl^-^

Performance gains via SOCl_2_ treatment of SWCNT-silicon solar cells have been reported previously [6,7]. The effect of SOCl_2_ on SWCNTs has been relatively well-studied [11,26,27,28,29] and is similar to HNO_3_ doping [19,47]. SOCl_2_ has been shown to be a very good *p*-type dopant for SWCNTs that increases conductivity in the SWCNTs by shifting the Fermi level into the valance band (as evidenced in this study by the bleaching of S_11_ absorption), and in the films by reducing barriers at the tube-tube junctions [44,47]. After SOCl_2_ treatment, the conduction mechanism of the SWCNT films switches from variable range hopping/thermionic emission over tube-tube barriers to tunneling through them [8,44].

**Figure 6:**
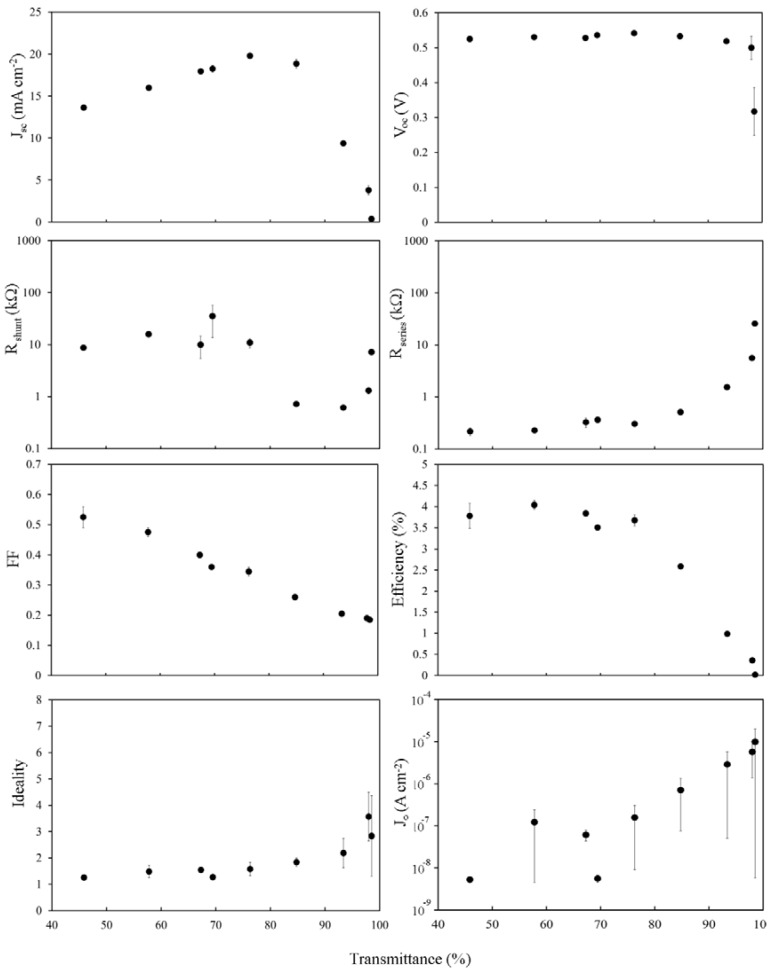
Solar cell parameters extracted from SOCl_2_ treated SWCNT-Si devices with varying SWCNT film thickness.

The *J*_sc_ peak in Figure 6 has shifted to higher transmittance (~65% to ~80%) in line with the change observed in *T*_th_ (Figure 2b) as well as the magnitude of *J*_sc_ at each film thickness increasing to ~125% of the undoped value. The insensitivity of *V*_oc_ to film thickness (except for the 98% film) has returned to exactly as it was before the HF treatment whilst the fill factors have maintained or improved upon their overall gains compared to the as prepared case. There has been a dramatic change in the behavior of *R*_shunt_, with two distinct regions now separated by a sharp break at ~80%, but *J*_0_ and the ideality remain the same.

The increases in *J*_sc_ and *V*_oc_, as well as modest improvement in *FF* and *R*_s_, coupled with the large improvement of *R*_shunt_ for 55% < *T*_av_ < 80%, lead to significant PCE gains of up to 350% of the undoped values (for *T*_av_~75%) and the pattern of variation in PCE is now clearly due to limited *R*_s_.

### 2.5. The Effect of a Second HF Treatment, with 1 h or 4 h Oxide

**Figure 7:**
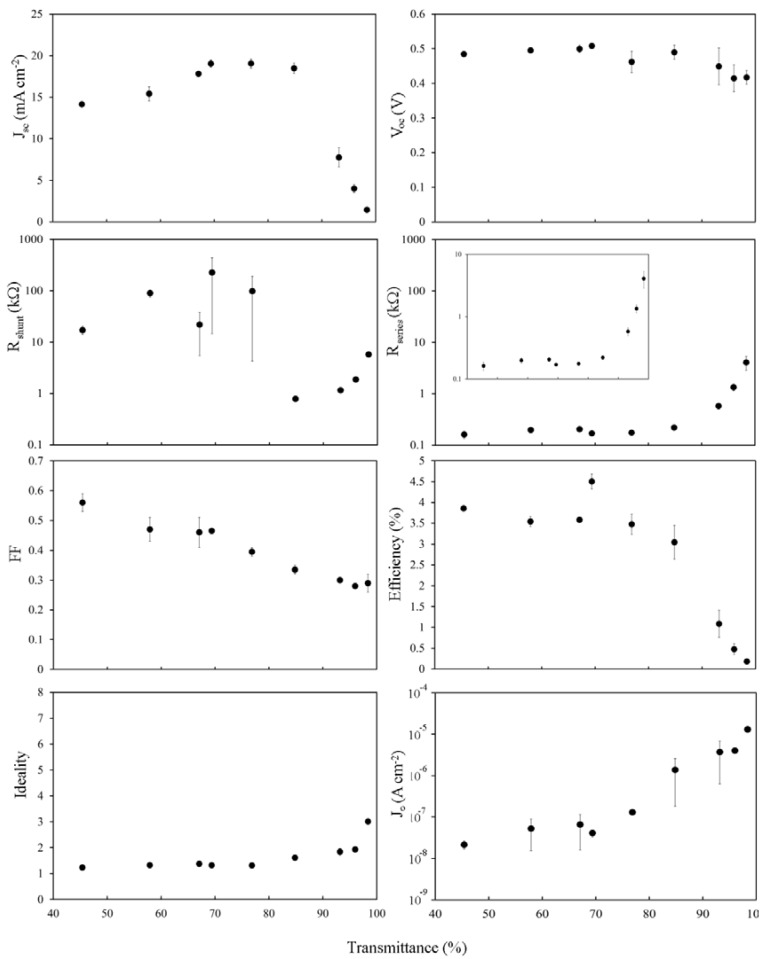
Solar cell parameters extracted from HF treated (second treatment) SWCNT-Si devices with varying SWCNT film thickness, 1 h after treatment.

Following the SOCl_2_ treatment, a second brief HF treatment improves the fill factor (Figure 7), particularly for the thinner films, and this is likely due to lower *R*_s_. *V*_oc_ has been reduced by ~10% but is now reasonably high even for the device with the 98% film. *J*_sc_ and *J*_0_ are unchanged but the ideality is modestly improved in line with *R*_s_ for the thinner films. *R*_shunt_ is markedly improved in the devices with the thicker films but unchanged for *T*_av_ > 80%. It has been shown that the effect of acid doping on the conductance of SWCNT films sensitively depends on the film thickness [48] and a similar phenomenon exists here. The threshold at which *R*_s_ begins to increase rapidly has not increased as much as for *R*_sheet_, and did not for the SOCl_2_ case either, but this is consistent with a model in which the percolation factor (*R*_junction_) contributing to *R*_s_ has an additional term that *R*_sheet_ does not, representing the dependence on the number of the SWCNT-silicon junctions.

The PCE of these particular devices remained relatively unchanged by the second HF treatment but it was often observed that this step was critical in attaining the best performance from devices following the SOCl_2_ treatment. A third HF treatment immediately following the second had no noticeable effect however, after multiple consecutive HF treatments, there comes a point where the undercutting of the oxide underneath the front gold electrode causes collapse of this onto the silicon and results in a very noticeable drop in performance. If left for several weeks, the performance of all devices returned to about the same as the as prepared state.

**Figure 8:**
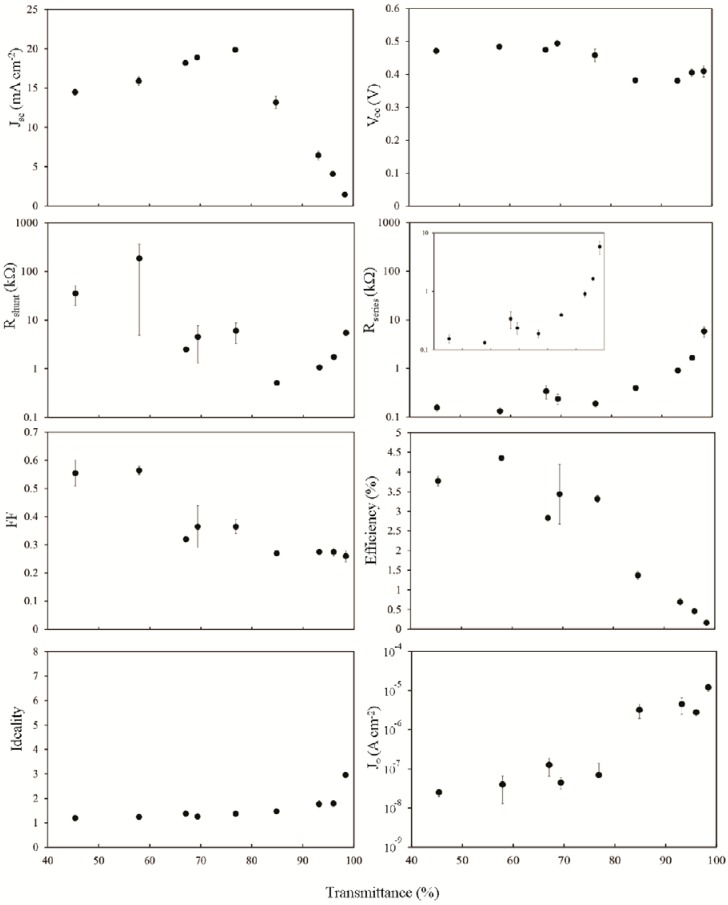
Solar cell parameters extracted from HF treated (second treatment) SWCNT-Si devices with varying SWCNT film thickness, 4 h after treatment.

After 4 h in ambient laboratory air, there will certainly be a native oxide (Figure 8). The overall shape of *J*_sc_ is again unchanged and still peaks at ~75%–80% but the fill factor, *R*_s_, *R*_shunt_ and PCE have all been affected to some degree, predominantly in the middle of the thickness range. The growth and removal of an interfacial oxide has been shown to reversibly improve the performance of some randomly aligned SWCNT-silicon solar cells [21,49] but this is not true of all reports.

Jung reasons that the operating mechanism in their solar cells, made with highly aligned SWCNT films, is not that of a MS Schottky junction primarily because the very high activation energy, *E*_a_ is equal to *E*_g_, the energy gap of silicon (1.1 eV) and is far too high for a Schottky barrier, and because of the very long minority carrier lifetime in the microseconds [20]. It is also reasoned that the mechanism of action is the same as in *p*-*n* not MIS solar cells because the growth of a thin oxide does not improve their devices’ performance, contrary to the observations of Jia [21,49].

The best of SWCNT films reported here (~1 kΩ·sq^−1^ for *T*_av_ = 90%) are certainly better than many earlier examples such as the high density films in early reports by Li [8] (10–100 kΩ·sq^−1^ for *T*_550_ = 90%) but are outperformed by the latest in very highly aligned films (~10 Ω·sq^−1^ for *T*_550_ = 90%) [31] using a modification of the super acid method of Saha [50].

Consistent with the results shown here, it may be that the thin oxide is required in between the sparse SWCNT junctions formed by randomly aligned films to prevent recombination at these uncovered surfaces and allow carriers time to find a nearby SWCNT-silicon junction in the imperfectly covering film. The ability of the devices to separate charge would thus be governed primarily by the Si:SiO*_x_* junction, hence the relative insensitivity of *V*_oc_ towards SWCNT film thickness in the presence of the oxide and the *E*_a_ = *E*_g_ observation. This is unnecessary with the very low resistance aligned films and the conformal covering they provide.

## 3. Experimental Section

### 3.1. Preparation of SWCNT Suspension

Arc-discharge SWCNTs (5 mg, P3-SWCNT, Carbon Solutions Inc., Riverside, CA, USA) were bath sonicated at ~50 W_RMS_ for 1 h in an aqueous solution of TritonX-100 (50 mL, 1% *v*/*v*, Sigma, Castle Hill, Australia). The resulting suspension was centrifuged in 6 × 8 mL tubes for 1 h at 17,500 g. This first supernatant was collected, combined and then centrifuged for a further 1 h at 17,500 g. The upper 6 mL of the second supernatant was collected and combined to yield a (very slightly red) black suspension (36 mL). The centrifuge residues were combined, filtered onto a pre-weighed polycarbonate membrane (0.45 μm, HTTP, Millipore, Kilsyth, Victoria, Australia), rinsed thoroughly with 250 mL of deionized (DI) water and then dried in air for 1 h at 80 °C. The final mass of the unwanted residue was 3.2 mg, giving a process yield (into the suspension) of 36%. The yield can be improved by using a high powered tip sonicator however the bath sonicator was used to minimize damage to the SWCNTs.

### 3.2. SWCNT Film Formation by Vacuum Filtration

Randomly aligned SWCNT membranes were prepared similarly to Wu [15] and Hu [37]. Volumes of 25, 50, 75, 150, 225, 300, 375, 450 or 600 μL·cm^−2^ of filter membrane of the SWCNT suspension was first diluted in 250 mL of a 0.01% *v*/*v* solution of TritonX-100 in DI water and then filtered onto large pore mixed cellulose ester (MCE) “target” membranes (0.45 µm, HAWP, Millipore, Kilsyth, Victoria, Australia) over a smaller pored “stencil” membrane (25 nm, VSWP, Millipore, Kilsyth, Victoria, Australia). The very large difference in flow rates between target and stencil allows the fabrication of well-defined film shapes on the target membrane, all with identical characteristics. The films made this way are highly reproducible and the thickness/optical density are precisely controllable by varying the concentration and/or volume of SWCNT solution, with the additional benefit of minimizing wastage of SWCNT material. However, to get good long term reproducibility and control of films’ optical and sheet resistance characteristics, it was found that the same apparatus (glass filter frit, *etc.*) must be used with the same vacuum applied and the concentration of surfactant and age/bundle state of the suspension must all be well controlled. The SWCNT films were rinsed thoroughly with 3 × 50 mL DI water then a further 250 mL DI water. Circular regions (0.32 cm^2^) of the resulting SWCNT-MCE membrane were taken for device fabrication.

### 3.3. Device Fabrication

Phosphorous doped *n*-type silicon wafers (CZ, 5–25 Ω·cm, <100>, SSP, ABC GmbH, München, Germany) with a thermal oxide (100 nm) were diced into rectangular pieces (1 × 1.5 cm^2^). UV photolithography was used to define circular regions (0.08 cm^2^) in a positive resist (AZ1518, micro resist technology GmbH, München, Germany), which was developed and then the front metal contact (Ti/Au, 5/145 nm) was sputtered.

Following photoresist lift off the 100 nm oxide in these regions was removed with buffered oxide etch (BOE). The SWCNT films were deposited onto the substrate surfaces by placing the circular SWCNT-MCE membranes, SWCNT side down, centered over the etched holes. The membranes were wetted with DI water then compressed and baked dry (80 °C, 15 min). Following cooling, the substrates were immersed in acetone for 1 h to dissolve the MCE. After being removed from this first acetone bath and dried with N_2_, the devices were washed in a further 3 baths of fresh acetone for 1 h each and with mild stirring of the solvent. After the final drying with N_2_, substrates were obtained with SWCNT membranes tightly adsorbed onto their surfaces providing 0.08 cm^2^ circular active areas surrounded by 0.24 cm^2^ regions where the SWCNT membranes overlap the front metal contacts. After etching of the rear oxide the devices were completed with gallium indium eutectic (eGaIn) back contacts and mounted on steel plates (2 cm^2^). The solar cells, shown schematically in Figure 3, thus produced are called “as-prepared”.

### 3.4. Characterization

UV-vis-NIR absorbance spectra of the SWCNT films were measured using a spectrophotometer (Cary50, Varian, Mulgrave, Victoria, Australia) by passing the beam through films mounted on glass. Simple background subtraction was performed using the absorbance spectra of the glass substrates. Transmittance spectra were calculated post-measurement from the absorbance data. Sheet resistance measurements were taken from the same films using a four point probe (KeithLink) in linear geometry and a multimeter (GDM-8261, GW Instek, Lepas, Penang, Malaysia).

To calculate a reliable measure of the average optical depth the absorbance was averaged from two points chosen so as to be outside the range of the SWCNT absorption peaks. This is because those regions are subject to large changes due to post fabrication film treatments, which would skew the absorbance values. For these large diameter arc-discharge SWCNTs the two wavelengths were 450 nm and 850 nm and the absorption was converted to an average transmittance.

Current-voltage data was collected with a Keithley 2400 source measure unit and recorded using a custom Labview™ virtual instrument. For light experiments, the cells were illuminated by collimated 100 mW·cm^−2^ light from a xenon-arc source passed through an AM1.5G filter. The irradiance at the sample plane was measured with a silicon reference cell (PV Measurements, NIST-traceable calibration, Denver, CO, USA). Data was obtained by scanning from 1 V to −1 V and several curves were taken to verify the stability of the output characteristics.

The solar cell parameters *J*_sc_, *V*_oc_, *FF*, and PCE were determined and the series resistance (*R*_s_) and shunt resistance (*R*_shunt_) were estimated from the inverse slopes of the current-voltage curves under illumination at *V*_oc_ and *J*_sc_, respectively. The dark current characteristics were analyzed using the diode Equation [51]:

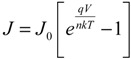
(1)
where *J*_0_ is the reverse saturation current density, *V* the applied potential; *q* the elementary charge; *k* the Boltzmann constant; *T* the absolute temperature; and *n* the ideality factor. For *V* > *kT*/*q*, the −1 term can be dropped since the exponential term rapidly becomes >>1. Taking the natural logarithm of both sides gives:

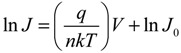
(2)


Thus, the semilog plot of the current-voltage data (ln*J versus V*) yields *n* and *J*_0_ from the slope and intercept, respectively. This method works well for close-to-ideal diodes however in some of the SWCNT-silicon test cells the voltage drop due to high parasitic *R_s_* cannot be ignored. Thus, Equation (1) can be modified to give [52]:

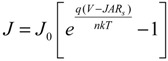
(3)
where *A* is the active area of the junction and *R*_s_ is the combined series resistances of the device. In this case a plot of ln*J versus* (*V* − *JAR*_s_) can be more accurate, assuming a reliable measure of *R_s_* can be made. It can be estimated from the light *JV* data but possible light-induced effects must be taken into account. Another way to determine *R*_s_ (but which assumes a Schottky diode [51]) is to take the logarithm of Equation (3), differentiate with respect to *J* and rearrange terms to give:

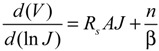
(4)
where β = *kT*/*q* and a plot of *d*(*V*)/*d*(ln*J*) *versus J* yields the series resistance and a self-consistency check of the ideality factor from the slope and intercept, respectively. Regardless, as with all modeling of real data, it must be done with a good idea of what should be expected and an understanding of the limitations and alternatives of the model used.

### 3.5. Film Treatments Applied Post Fabrication

After characterization, the as prepared devices were treated for 15 s with 2% HF to remove the native oxide. This was done by placing a single drop on the film surface, which was readily wetted, and then rinsing with water and then ethanol. HF dissolves glass thus it was not possible to use it on the glass-mounted films used for optical and sheet resistance measurements and so 2% HCl was used instead, in the same manner. For thionyl chloride (SOCl_2_) treatment, two drops were applied to the SWCNT films, allowed to dry in air, and then rinsed briefly with ethanol and dried with N_2_. A second HF treatment was applied in the same manner as the first.

## 4. Conclusions

The results show a strong dependence on film thickness, as expected, particularly for films with average transmittance greater than ~70% and the main cause of this is the sheet resistance of the SWCNT film. The effect of doping with SOCl_2_ is primarily via a reduction in sheet resistance boosting the short circuit current density, open circuit voltage and fill factor of all devices. An interfacial oxide does not improve the devices with doped SWCNT films, but is essential for those with the undoped films, and particularly the thinner films with *T*_av_ > 70%.
